# 2113. Infectious endocarditis in patients with hematologic malignancy at the University of Wisconsin

**DOI:** 10.1093/ofid/ofac492.1734

**Published:** 2022-12-15

**Authors:** Michael J Scolarici, Leigh R Berman, Christopher Saddler, Jeannina Smith

**Affiliations:** University of Wisconsin Hospitals and Clinics, Madison, Wisconsin; University of Wisconsin School of Medicine and Public Health, Madison, Wisconsin; University of Wisconsin Department of Medicine Division of Infectious Diseases, Madison, Wisconsin; University of Wisconsin Department of Medicine Division of Infectious Diseases, Madison, Wisconsin

## Abstract

**Background:**

Infective endocarditis (IE) is a highly morbid complication of blood stream infections (BSIs) involving neutrophil and platelet activation. Patients with hematologic malignancies have frequent BSIs but a low incidence of IE. We sought to evaluate the microbiology of BSIs and rates of IE in hospitalized patients with hematologic malignancies.

**Methods:**

Patients admitted to the University of Wisconsin Hospital hematology ward between 2018 and 2020 were included if blood cultures obtained grew *Streptococcus spp., Staphylococcus spp., Enterococcus spp.,* Streptococcus-like bacteria, and fungi. Two investigators recorded patient demographics, BSI microbiology, and hospital course. Using the Duke’s criteria each BSI was classified as definite, possible, or rejected cases of IE. Cases of possible IE were reviewed for evidence of missed IE within 90 days of hospital admission based on readmission with repeat positive blood culture and new signs or symptoms of IE. Definite cases were reviewed by two additional investigators to corroborate cases of IE.

**Results:**

Our study included 101 unique patients with hematologic malignancy: 35.6% female, mean age 58.4, and 10.9% had multiple BSIs during our study period. Of 111 distinct BSIs, 82.6% were neutropenic, 37.4% community acquired, and 21.7% polymicrobial. Of 129 positive blood cultures, 41.0% isolated *Streptococcus spp.*, 5.4% *Staphylococcus aureus*, 17.8% other *Staphylococcal spp*., 7% *Enterococcus faecalis*, 9.3% other *Enterococcus spp*., 9.3% *Gamella spp.,* and 4.7% *Candida spp.* Of 111 BSI, 2 were classified as definite cases, 39 as possible, 70 as rejected. After further review, only 1 was considered a true cases of IE and they were not neutropenic. The 90-day survival rate for all patients was 76.2%.

**Conclusion:**

We found a very low incidence of IE in our study. Low rates of IE in our population aligns with previous reports showing rare cases of IE in neutropenic and malignancy patients with BSIs and raises questions about the utility of invasive IE workups in neutropenic patients with hematologic malignancies. Further studies are needed to identify patient or microbiologic factors that can be used to guide appropriate use of transesophageal echocardiography to evaluate IE in patients with hematologic malignancy.

**Disclosures:**

**All Authors**: No reported disclosures.

